# STING Degradation by PRRSV Activates HK_2_-Mediated Glycolysis to Facilitate Viral Replication

**DOI:** 10.3390/v18030284

**Published:** 2026-02-27

**Authors:** Li Luo, Long Zhou, Xue Gao, Yuling Li, Han Zhou, Yanmin Li, Zhidong Zhang

**Affiliations:** Key Laboratory of Animal Medicine of Sichuan Province, College of Animal and Veterinary Sciences, Southwest Minzu University, Chengdu 610093, Chinaliyanmin@swun.edu.cn (Y.L.)

**Keywords:** PRRSV, STING, HK_2_, glycolysis, viral replication

## Abstract

Porcine reproductive and respiratory syndrome virus (PRRSV) infection relies on glycolytic reprogramming to support replication, but the mechanisms driving this metabolic shift remain poorly understood. The stimulator of interferon genes (STING), an innate immune adaptor, recently emerged as a metabolic regulator by directly binding and inhibiting hexokinase-2 (HK_2_), a key rate-limiting enzyme in glycolysis. Whether PRRSV exploits the STING-HK_2_ axis to unleash glycolysis for its own replication is unknown. Here we demonstrate that PRRSV infection induced STING degradation and promoted HK_2_ suppression, activating glycolysis for viral replication. In PRRSV-infected Marc-145 cells, lactate production (a glycolysis marker) and HK_2_ expression increased time-dependently, peaking at 48 h post-infection (hpi). Conversely, STING protein levels decreased significantly at 36 hpi and further at 48 hpi, suggesting a correlation between STING downregulation and glycolytic activation. The HK_2_ inhibitor 2-deoxy-D-glucose reduced lactate production and viral load, while the glycolysis activator PS48 enhanced both. STING knockdown via siRNA increased HK_2_ expression, lactate secretion, and PRRSV nucleocapsid protein levels, whereas STING overexpression suppressed these phenotypes. Co-immunoprecipitation and confocal microscopy demonstrated direct STING-HK_2_ interaction and cytoplasmic co-localization, maintained during PRRSV infection. HK_2_ overexpression promoted viral replication without altering STING levels, confirming HK_2_ as a downstream effector. In conclusion, PRRSV-triggered degradation of STING enhances HK_2_ expression, promoting lactate accumulation and accelerating viral replication. These findings suggest that the STING-HK_2_ axis can act as a critical viral metabolic checkpoint and highlight targeting metabolic–immune crosstalk as a potential anti-viral strategy.

## 1. Introduction

Porcine reproductive and respiratory syndrome virus (PRRSV) remains an economically devastating pathogen in the global swine industry. Since its first description in the United States in the late 1980s, PRRSV has spread worldwide [1]. PRRSV belongs to the family *Arteriviridae* and possesses a positive-sense single-stranded RNA genome approximately 15.4 kb in length. Based on genomic sequence identity, PRRSV is categorized into two species (PRRSV1 and PRRSV2). The viral genome consists of 11 open reading frames (ORFs), which encode 16 nonstructural proteins and eight structural proteins [2]. PRRSV’s extensive genetic variability complicates vaccine development and disease control. PPRSV infection can cause reproductive failure in pregnant sows and respiratory distress in pigs of all ages, often accompanied by secondary infections due to immunosuppression associated with PRRSV infection [3,4].

A defining feature of PRRSV pathogenesis is its ability to reprogram host cellular metabolism to support viral replication. Like many viruses, PRRSV upregulates glycolysis to meet heightened energy and biosynthetic demands. Lactate, traditionally considered a passive glycolytic end-product, now emerges as an immunometabolic signal which can suppress anti-viral immunity [5,6]. Recent studies have shown that PRRSV-induced lactate accumulation suppresses innate immunity by lactylating MAVS, thereby dampening RIG-I-like receptor signaling, creating a creating a self-reinforcing loop that facilitates viral replication while dampening immunity [7,8]. However, the upstream molecular triggers of this glycolysis remain poorly understood.

The stimulator of interferon genes (STING) is a conserved innate immune adaptor which is not only known for its canonical role in sensing cytosolic DNA and inducing type I IFN responses [9] but also its vital role in the host innate immune response against RNA viral infections and RNA virus replication, including PRRSV [10]. Emerging evidence, however, reveals STING as a metabolic regulator that directly binds and inhibits hexokinase-2 (HK_2_), the rate-limiting enzyme of glycolysis that catalyzes the first committed step of glycolysis (phosphorylating glucose to glucose-6-phosphate). The STING-HK_2_ interaction constrains aerobic glycolysis in cancer cells and promotes anti-tumor immunity in vivo [11]. For PRRSV, which relies on glycolysis for replication, STING could act as a metabolic barrier by restricting HK_2_ activity. PRRSV is known to employ immune evasion strategies by targeting STING. PRRSV inhibits STING-induced innate immunity by blocking the translocation of STING from the endoplasmic reticulum (ER) to the Golgi apparatus, blocking IFN-β induction [12], and the viral nsp5 protein deters STING translocation and activation to suppress the innate anti-viral response [13]. However, whether PRRSV additionally manipulates STING’s metabolic function to unleash glycolysis remains unexplored. STING’s ability to inhibit HK_2_ suggests a potential mechanism by which PRRSV may disrupt STING-HK_2_ interaction, and HK_2_ could be derepressed to drive glycolysis which in turn upregulates lactate production for facilitating its own replication. To address this, we analyzed the STING expression change during infection and investigated the regulatory link between PRRSV infection and HK_2_ induction.

In the present study, we revealed that PRRSV infection triggered degradation of STING to upregulate HK_2_ expression, leading to lactate production and facilitating viral replication. Targeting the STING-HK_2_ axis may provide a host-directed strategy to restrict PRRSV. These findings not only advance PRRSV pathogenesis but also highlight STING as a metabolic checkpoint vulnerable to viral manipulation, with implications for other glycolysis-dependent viruses. Ultimately, targeting this axis may offer a strategy to combat PRRS and reduce the global economic burden of swine diseases.

## 2. Materials and Methods

### 2.1. Antibodies

The antibodies used in this study included anti-rabbit STING (50494S; Cell Signaling Technology, Danvers, MA, USA), anti-Flag (HA722780; HUABIO, Hangzhou, China), anti-GFP (ET1607-31; HUABIO,), anti-PRRSV-N (GTX642546; GeneTex, San Antonio, TX, USA), anti-β-tubulin (GB152667-100; Servicebio, Wuhan, China), and anti-mouse HK_2_ (66974-1-Ig, Proteintech, Rosemont, IL, USA). Secondary antibodies included goat anti-rabbit IgG-HRP antibody (31460; Invitrogen, Waltham, MA, USA), goat anti-mouse IgG-HRP (31430; Invitrogen,) and Alexa Fluor 594-conjugated goat anti-mouse IgG (HA1126; HUABIO).

### 2.2. Cell and Virus

PRRSV-permissive Marc-145 cells were maintained in Dulbecco’s modified Eagle’s medium (DMEM) (G4524, Servicebio, Wuhan, China) containing 10% fetal bovine serum (FBS) (C2510, Viva Cell, Shanghai, China) and 1% penicillin–streptomycin. The cells were cultivated in a humidified incubator in a 5% CO_2_ atmosphere and at 37 °C.

The PRRSV strain SC14XC-1 (GenBank accession number KT03050) was isolated in 2014 and has been maintained in our laboratory. The virus was propagated and titrated in Marc-145 cells cultured in DMEM supplemented with 2% fetal bovine serum (FBS). Viral titers were determined using the Reed–Muench method and expressed as the 50% tissue culture infectious dose (TCID_50_).

### 2.3. Lactate Measurement

Extracellular lactate concentration was measured by a Lactic Acid Assay kit (Cat No. A019-2-1, NanjingJiancheng Bioengineering, China, according to the manufacturer’s instructions. Absorbance was measured at a wavelength of 530 nm.

### 2.4. Transfection and Gene Silencing with siRNAs

The siRNAs targeting monkey STING (GenBank accession: MF622060.1) were custom-designed and synthesized by Gene Create (Wuhan, China) and the siRNA sequences are listed in Table 1. Marc-145 cells were seeded in 6-well plates and transfected with 100 nM siRNA using the Lipo8000™ transfection reagent (C0533; Beyotime, Shanghai, China) when the cells reached 70–80% confluency, following the manufacturer’s instructions. To assess knockdown efficiency, total RNA was extracted from the cells at 48 h, and endogenous STING mRNA levels were quantified by RT-qPCR. In parallel, cells were lysed, and STING protein expression was measured by Western blotting using an anti-STING rabbit antibody (1:1000).

### 2.5. Plasmid Transfection

pEGFP-STING and HK_2_-Flag were custom-designed and synthesized by Gene Create (Wuhan, China). For transfection, the Marc-145 cells were grown in culture dishes to approximately 70% confluence. Plasmids (2 μg) were transfected into the cells using the Lipo8000™ transfection reagent (C0533; Beyotime) according to the manufacturer’s instructions.

### 2.6. Drug Treatment

The Marc-145 cells were treated with or without 10 mM of 2-deoxy-D-glucose (HY-13966; Med ChemExpress, Monmouth Junction, NJ, USA) and 20 μm PS48 (HY-15967, MedChemExpress, Monmouth Junction, NJ, USA) 2 h after infection with PRRSV (MOI = 1). 2-Deoxy-D-glucose is a glucose analog that serves as a competitive inhibitor of glucose metabolism, specifically inhibiting glycolysis by interfering with hexokinase activity. PS48 is a PDK1 activator that promotes the shift of glucose metabolism from the TCA cycle to glycolysis.

### 2.7. RT-qPCR

Total RNA was extracted using TRIzol reagent (RM0101; AccuRef Scientific, Xian, China). The extracted RNA was reverse-transcribed into cDNA using ReverTra Ace qPCR RT Master Mix with gDNA Remover (FSQ-301; Toyobo, Osaka, Japan). Quantitative real-time PCR was performed using PowerUp™ SYBR™ Green Master Mix (A25742; Thermo Fisher, Waltham, MA, USA). The primer pair PRRSV-F (5′-CAGGGTGCTGGAACTTGTGC-3′) and PRRSV-R (5′-GCTGAGGGTGATGCTGTGGC-3′) was used for amplification. A recombinant plasmid containing the PRRSV ORF7 gene was used to generate a standard curve for the quantification of viral RNA copy numbers in different samples.

### 2.8. Western Blotting

Cells were harvested and lysed in RIPA lysis buffer (R0020; Solarbio, Beijing, China) supplemented with the protease inhibitor PMSF (No. 2307001; Solarbio). The lysates were centrifuged, and the supernatants were mixed with 2× SDS sample buffer (abs9237; Absin, Shanghai, China). Proteins were separated by 12% SDS–PAGE and transferred onto polyvinylidene difluoride (PVDF) membranes (Millipore, Burlington, MA, USA). The membranes were blocked with 5% nonfat milk at room temperature for 3 h, cut according to the molecular weight of the target proteins, and then incubated with primary antibodies overnight at 4 °C. After washing, the membranes were incubated with horseradish-peroxidase-conjugated secondary antibodies at room temperature for 2 h. Protein bands were visualized using an enhanced chemiluminescence (ECL) detection system (G2020; Servicebio) and imaged with a BLT GelView6000Plus imaging system.

### 2.9. Co-Immunoprecipitation

Marc-145 cells were transfected individually or co-transfected with STING and HK_2_ expression plasmids for 48 h, followed by co-immunoprecipitation (Co-IP) using the Pierce™ Classic Magnetic IP/Co-IP Kit (P2175S; Beyotime). Cells were lysed in an appropriate amount of lysis buffer at 4 °C for 20 min and centrifuged at 13,000× *g* for 10 min, after which the supernatants were collected. A small aliquot of each lysate was reserved as an input control, and the remaining lysate was incubated with 5–10 µg of primary antibodies overnight at 4 °C. Subsequently, 20 µL of Pierce Protein A/G magnetic beads was added to the antigen–antibody complexes and incubated at room temperature for 1 h. The beads were collected using a magnetic rack, washed three times, and eluted with elution buffer. Protein–protein interactions were analyzed by Western blotting.

### 2.10. Immunofluorescence and Confocal Microscopy

The Marc-145 cells were seeded in poly-L-lysine-coated confocal dishes. After adherence, the cells were transfected individually or co-transfected with the HK_2_ and STING incubated for 48 h. The cells were fixed with 4% paraformaldehyde (Beyotime, China) for 20 min, permeabilized with 0.2% Triton X-100 (Solarbio, China) for 15 min, and blocked with 5% bovine serum albumin (BSA) for 1 h. Subsequently, the cells were incubated with the primary antibody overnight at 4 °C, followed by incubation with the appropriate fluorescent-dye-conjugated secondary antibody for 1 h at 37 °C in the dark. Finally, nuclei were counterstained with DAPI (ab104139, Abcam, Cambridge, UK) for 5 min.

### 2.11. Statistical Analysis

The data were processed with Microsoft Excel and GraphPad Prism 8.0 and are presented as mean ± SD of one representative experiment. Student’s *t*-test, two-sided, unpaired, two-tailed, and two-way or one-way analysis of variance (ANOVA) were used to analyze data as indicated. *p* < 0.05 was considered statistically significant.

## 3. Results

### 3.1. PRRSV Infection Promotes Glycolysis via Upregulation of HK_2_ Expression

**Figure 1:**
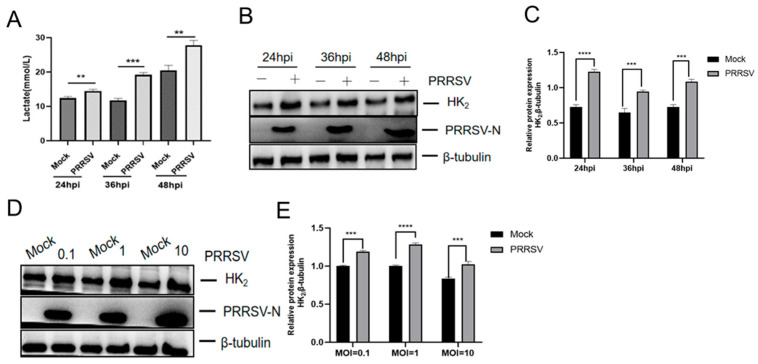
PRRSV infection induces glycolysis by upregulating HK_2_ expression. (**A**) PRRSV infection induces lactate production. (**B**) PRRSV infection upregulates HK_2_ expression as determined by Western blotting. (**C**) HK_2_ protein levels, normalized to β-tubulin, were quantified by densitometric analysis. (**D**) HK_2_ protein levels in cells infected with different MOIs of PRRSV determined by Western blotting. (**E**) HK_2_ protein levels, normalized to β-tubulin, in cells infected with different MOIs of PRRSV were quantified by densitometric analysis. Data are presented as mean ± SD. **, *p* < 0.01; ***, *p* < 0.001; ****, *p* < 0.0001.

To determine whether PRRSV infection influences glycolytic activity, we first measured lactate production, a key indicator of glycolysis, in the culture supernatants of PRRSV-infected Marc-145 cells using a Lactic Acid Assay kit. As demonstrated in Figure 1A, lactate levels were significantly elevated in infected cells at 24, 36, and 48 h post-infection (hpi) compared to uninfected control cells (*p* < 0.01), with the highest level observed at 48 hpi. These results confirmed that PRRSV infection induces glycolytic activation. To further corroborate the effect of PRRSV infection on the glycolysis, we assessed the expression of HK_2_, a key rate-limiting enzyme in glycolysis, in infected cells. Western blot analysis demonstrated that HK_2_ expression was upregulated from 24, 36, and 48 hpi (*p* < 0.001) compared to uninfected control cells (Figure 1B,C) and increased in a dose-dependent manner (Figure 1D,E). Collectively, these data indicate that PRRSV infection triggers glycolysis through the upregulation of HK_2_.

### 3.2. HK_2_-Mediated Glycolysis Is Critical for PRRSV Replication

Proteomic profiling of PRRSV-infected cells previously identified glycolysis-associated enzymes as potential regulators of viral replication [14]. To dissect the role of HK_2_ specifically, we first modulated glycolytic flux using pharmacological treatment.

**Figure 2:**
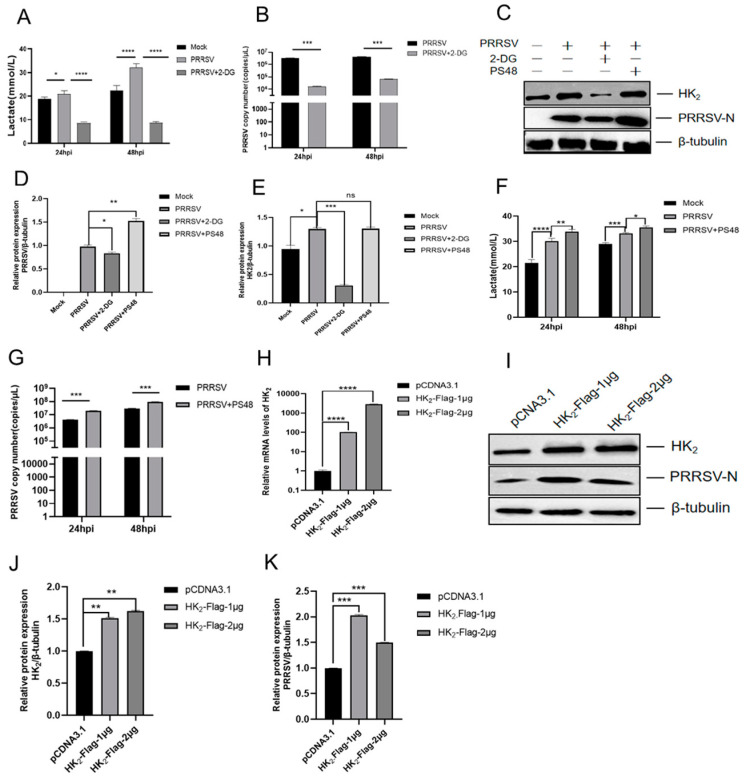
HK_2_ facilitates PRRSV replication. (**A**,**F**) Marc-145 cells were inoculated with PRRSV (MOI = 1) for 2 h and then treated with 20 nM 2-DG or 10 μM PS48. Lactate concentrations in the culture supernatants were measured using a Lactic Acid Assay kit at 24 and 48 hpi. (**B**,**G**) Marc-145 cells were handled under the same treatment and infection conditions as in (**A**), and viral RNA levels were quantified by qRT-PCR. (**C**) Marc-145 cells were handled under the same treatment and infection conditions as in (**A**), and samples were collected at 48 hpi, PRRSV-N and HK_2_ expression was detected by Western blotting. (**D**,**E**) PRRSV-N and HK_2_ protein levels, normalized to β-tubulin, were quantified by densitometric analysis. (**H**) HK_2_ overexpression efficiency. (**I**) HK_2_ overexpression reduces PRRSV-N expression in PRRSV-infected cells. (**J**,**K**) PRRSV-N and HK_2_ protein levels, normalized to β-tubulin, were quantified by densitometric analysis. Data are presented as mean ± SD. *, *p* < 0.05; **, *p* < 0.01; ***, *p* < 0.001; ****, *p* < 0.0001.

HK_2_ inhibition suppresses PRRSV replication: Marc-145 cells were treated with 2-deoxy-D-glucose (2-DG; 10 mM), a competitive inhibitor of HK_2_ that blocks glycolysis by binding to HK_2_’s active site. Compared to untreated PRRSV-infected cells, 2-DG treatment significantly reduced lactate secretion at 24 hpi (*p* < 0.0001) and 48 hpi (*p* < 0.0001) (Figure 2A) concurrent with decreased viral RNA levels (*p* < 0.001) (Figure 2B) and PRRSV nucleocapsid (N) protein expression (*p* < 0.05) (Figure 2C,D) and HK_2_ expression (*p* < 0.001) (Figure 2C,E).

Glycolysis activation enhances PRRSV replication. Marc-145 cells were treated with a glycolysis activator. As opposed to untreated PRRSV-infected cells, treatment with PS48 (20 μM) significantly increased lactate secretion in PRRSV-infected cells at 24 hpi (*p* < 0.01) and 48 hpi (*p* < 0.05) (Figure 2F), along with elevated viral RNA levels (*p* < 0.01) (Figure 2G), PRRSV-N protein levels (*p* < 0.01) and HK_2_ expression (Figure 2C–E).

HK_2_ overexpression promotes PRRSV replication; to confirm that HK_2_ itself is sufficient, Marc-145 cells were transfected with either an HK_2_ overexpression plasmid (1 μg or 2 μg) or an empty vector as a control. RT-qPCR verified dose-dependent HK_2_ mRNA upregulation (1 μg: ~100-fold; 2 μg: ~3000-fold vs. control, *p* < 0.001) (Figure 2H), confirming effective activation of the transfected HK_2_ gene at the transcriptional level. Importantly, HK_2_ overexpression significantly increased PRRSV-N protein levels compared to empty vector controls (*p* < 0.01) (Figure 2I–K). These results demonstrate that PRRSV infection promotes HK_2_ upregulation, which in turn facilitates viral replication.

### 3.3. PRRSV Infection Induces STING Degradation to Promote HK_2_ Expression

Previous study indicated that STING could act as a metabolic barrier by directly binding and inhibiting HK_2_ expression [11]. To determine whether PRRSV targets STING to unleash HK_2_-driven glycolysis, we analyzed STING protein dynamics during infection and then performed STING knockdown and STING overexpression experiments.

**Figure 3:**
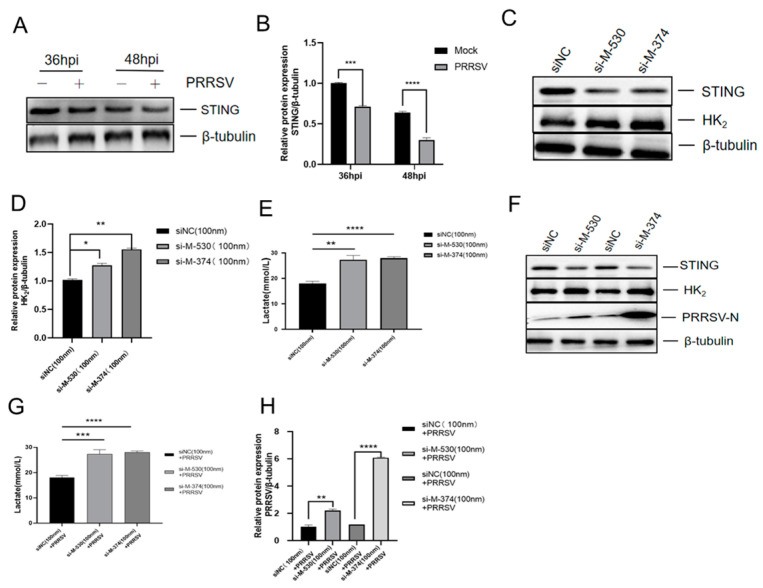
STING Knockdown promotes HK_2_ expression for replication. (**A**,**B**) PRRSV infection induces STING degradation. Marc-145 cells were inoculated with PRRSV (MOI = 1) for 36 h and 48 h, STING expression was detected by Western blot. (**C**,**D**) STING was silenced using specific siRNAs. Marc-145 cells were transfected with siSTING-374 and siSTING-530 (100 nM) or siNC (negative control siRNA, 100 nm) for 48 h. STING expression was analyzed by Western blotting. (**E**) Marc-145 cells were treated as described in (**C**), lactate concentrations in the culture supernatant were measured using a Lactic Acid Assay kit. (**F**,**G**) Marc-145 cells were treated as described in (**C**) for 12 h and then infected with PRRSV, lactate concentrations in the culture supernatant were measured using a Lactic Acid Assay kit. STING and PRRSV-N expression levels were determined by Western blotting. (**H**) PRRSV-N expression was quantified by densitometric analysis and normalized to β-tubulin. Data are presented as mean ± SD. *, *p* < 0.05; **, *p* < 0.01; ***, *p* < 0.001; ****, *p* < 0.0001.

PRRSV infection triggers STING degradation. Marc-145 cells were infected with PRRSV (MOI = 1) or mock-infected and harvested at 36 and 48 hpi. Western blot analysis revealed a time-dependent reduction in STING protein levels, with significant decreases at 36 hpi (*p* < 0.001) and 48 hpi (*p* < 0.0001) relative to uninfected controls (Figure 3A,B).

STING knockdown promotes HK_2_ expression and enhances PRRSV replication. We performed STING knockdown using two specific siRNAs (siSTING-374 and siSTING-530). Transfection with these siRNAs efficiently reduced STING mRNA by ~60% (siSTING-374) and by ~50% (siSTING-530) (*p* < 0.0001) (Figure S1A) and STING protein levels by ~60% (siSTING-374) and ~50% (siSTING-530) (*p* < 0.01) compared to scramble siRNA controls (Figure 3C and Figure S1B). STING knockdown significantly increased HK_2_ expression (*p* < 0.01) (Figure 3C,D) and lactate production (*p* < 0.01) (Figure 3E). In PRRSV-infected cells, STING silencing further enhanced HK_2_ expression by ~30% (siSTING-374) and by ~50% (siSTING-530) (*p* < 0.01) (Figure 3F) compared to the infected-scramble controls. Consistently, lactate production was also elevated (siSTING-374 *p* < 0.001 and siSTING-530 *p* < 0.0001) (Figure 3G). These metabolic changes correlated with enhanced PRRSV-N expression (Figure 3F–H), which was 2-fold higher in siSTING-374-transfected cells (*p* < 0.01) and 6-fold higher in siSTING-530-transfected cells (*p* < 0.0001).

**Figure 4:**
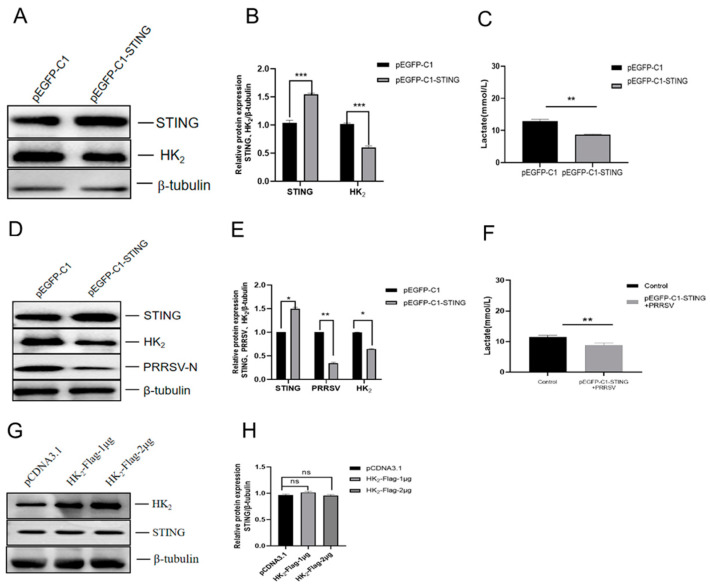
STING overexpression reduces HK_2_ expression and viral replication. (**A**,**B**) STING overexpression reduces HK_2_ expression. STING expression was detected with STING-specific antibody by Western blot. (**C**) STING overexpression reduces lactate production. (**D**,**E**) STING overexpression reduces HK_2_ expression and PRRSV-N expression in PRRSV-infected cells. (**F**) STING overexpression reduces lactate production in PRRSV-infected cells. (**G**,**H**) HK_2_ overexpression has no impact on STING expression. HK_2_ expression was detected with HK_2_-specific antibody by Western blot. Data are presented as mean ± SD. *, *p* < 0.05; **, *p* < 0.01; ***, *p* < 0.001.

STING overexpression suppresses HK_2_ expression and PRRSV replication. In cells transfected with the STING overexpression plasmid (pEGFP-STING), Western blot analysis with STING-specific antibody showed that HK_2_ expression levels (*p* < 0.01) (Figure 4A,B) and lactate production (*p* < 0.01) (Figure 4C) significantly decreased when compared to empty vector controls. When STING-overexpressing cells were infected with PRRSV, HK_2_ expression levels (*p* < 0.05) (Figure 4D,E) and lactate production (*p* < 0.01) (Figure 4F) remained significantly lower than in infected-control cells. These changes correlated with reduction in PRRSV-N protein expression (*p* < 0.01) (Figure 4D,E). These results demonstrate that STING negatively regulates glycolysis by inhibiting HK_2_ and that PRRSV infection degrades STING to relieve this inhibitory effect, thereby promoting glycolysis and facilitating viral replication. To clarify the regulatory relationship between STING and HK_2_, we examined whether HK_2_ overexpression affects STING expression. Results showed that HK_2_ overexpression detected with HK_2_-specific antibody did not alter protein levels of STING compared to controls detected STING-specific antibody (Figure 4G,H), indicating that STING acts upstream of HK_2_ in glycolytic regulation. The band observed in the empty vector control lanes corresponds to endogenous STING protein (Figure 4A,D) or HK_2_ protein (Figure 4G) due to use of STING or HK_2_-specific antibody. The grayscale analysis results clearly show a significant increase in protein expression in the transfected groups compared to the empty vector controls.

### 3.4. STING Interacts with HK_2_

**Figure 5:**
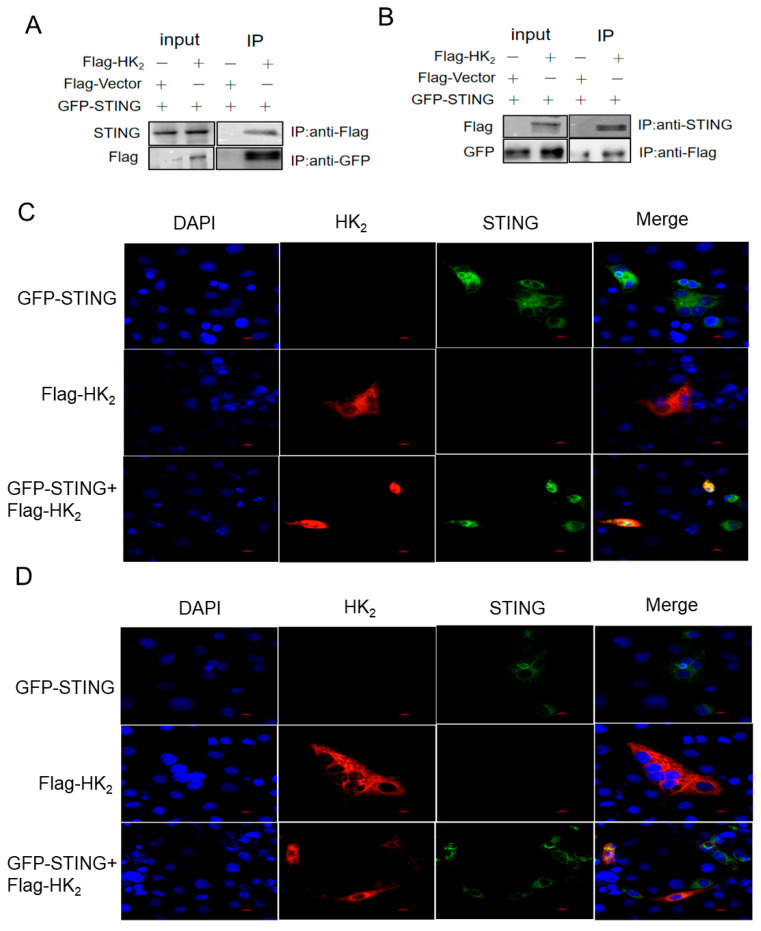
STING interacts with HK_2_. (**A**,**B**) Flag-tagged HK_2_ and GFP-tagged STING plasmids were transfected into Marc-145 cells either individually or in combination. Cell lysates were harvested and subjected to immunoprecipitation (IP) using mouse anti-Flag or mouse anti-GFP antibodies, followed by Western blot analysis with rabbit anti-Flag and rabbit anti-STING antibodies. (**C**) Subcellular localization of HK_2_ and STING. Plasmids expressing Flag-tagged HK_2_ and GFP-tagged STING were transfected into Marc-145 cells either individually or in combination for 48 h. Cells were fixed and subjected to indirect immunofluorescence assay using anti-Flag antibody (red)with nuclei counterstained with DAPI (blue). Green fluorescence indicates signal from the GFP tag, while yellow fluorescence represents the overlapping signals of anti-Flag antibody (red) and GFP (green). The upper panels: cells transfected with GFP-STING plasmid alone; the middle panels: cells transfected with Flag-HK_2_ plasmid alone; the lower panels: cells co-transfected with GFP-STING and Flag-HK_2_ plasmids. (**D**) Marc-145 cells were treated as described in (**C**) for 12 h and subsequently infected with PRRSV. The upper panels: cells transfected with GFP-STING plasmid alone; the middle panels: cells transfected with Flag-HK_2_ plasmid alone; the lower panels: cells co-transfected with GFP-STING and Flag-HK_2_ plasmids.

To investigate the mechanism by which STING regulates HK_2_, we performed co-immunoprecipitation (Co-IP) assays to examine their physical interaction. Marc-145 cells were transfected with Flag-tagged HK_2_ (HK_2_-Flag), GFP-tagged STING (GFP-STING), or co-transfected with both plasmids. Cell lysates were immunoprecipitated with anti-Flag or anti-GFP antibodies, followed by Western blot analysis. When immunoprecipitated with anti-Flag antibody, GFP-STING was co-precipitated with HK_2_-Flag in co-transfected cells but not in cells transfected with HK_2_-Flag alone. Conversely, when immunoprecipitated with anti-GFP antibody, HK_2_-Flag was co-precipitated with GFP-STING in co-transfected cells but not in cells transfected with GFP-STING alone (Figure 5A,B). These reciprocal Co-IP results demonstrate a specific physical interaction between STING and HK_2_. Furthermore, immunofluorescence confocal microscopy was performed to co-localize the STING and HK_2_ in uninfected Marc145 cells (Figure 5C) and PRRSV-infected cells (Figure 5D), respectively. The results showed that the STING expression (green) was observed in cells transfected with GFP-STING plasmid alone (Figure 5C upper panels), and HK_2_ expression (red) was seen in cells transfected with Flag-HK_2_ plasmid alone (Figure 5C middle panels). When cells were co-transfected with GFP-STING plasmid and Flag-HK_2_ plasmid, STING (green) and HK_2_ (red) were co-localized in the cytoplasm (Figure 5C lower panels), showing the overlapping signals of these two proteins in the same cellular compartment. This co-localization was maintained during PRRSV infection (Figure 5D lower panels). These results indicate that STING physically interacts with HK_2_ in the cytoplasm, supporting the model that STING directly inhibits HK_2_ function through protein–protein interaction.

## 4. Discussion

PRRSV remains a major threat to the global swine industry. There is increasing evidence indicating that viruses can hijack cellular metabolism to support replication [6,7,8,15,16,17,18]. PRRSV infection induces lactate production and promotes viral growth by suppressing IFN-β induction [7,8]. Lactate, historically regarded as a metabolic waste product, has recently been identified as a glycolytic metabolite which can inhibit RLR signaling by directly binding to the MAVS transmembrane domain and disrupting its aggregation [6,19]. However, the molecular mechanism underlying PRRSV-mediated lactate production remains unclear. In this study, we demonstrate that PRRSV degrades STING to promote HK_2_ expression, thereby activating glycolysis and enhancing viral replication. These findings reveal a novel regulatory axis involving STING-HK_2_-glycolysis that bridges innate immunity and cellular metabolism during PRRSV infection, providing new insights into viral–host interactions.

STING is well characterized as a key adaptor protein in innate immune signaling pathway, which mediates type I IFN responses against viral infections [9,20]. However, recent studies have uncovered its role as a metabolic regulator: STING directly binds to and inhibits HK_2_ to suppress glycolysis [11]. Our results extend this paradigm by showing that STING functions as a negative regulator of glycolysis during PRRSV infection. Specifically, STING knockdown via siRNA significantly increased HK_2_ expression and lactate production (Figure 3C–E), while STING overexpression exerted the opposite effects (Figure 4A–C). These results align with previous reports that STING inhibits cancer cell proliferation by suppressing HK_2_-mediated glycolysis [11], highlighting a conserved role for STING in metabolic checkpoint control across physiological and pathological contexts. Notably, PRRSV infection downregulated STING in a time-dependent manner (Figure 3A,B), suggesting that the virus targets STING not only to dampen innate immunity but also to manipulate cellular metabolism. This dual targeting strategy is reminiscent of other viruses, such as Peste des petits ruminants virus (PPRV) and Seneca Valley virus (SVV), which disrupt STING signaling to support replication [21,22]. However, our study demonstrated there is link between STING degradation and metabolic reprogramming, uncovering a new mechanism by which PRRSV subverts host defenses.

HK_2_ catalyzes the first and rate-limiting step of glycolysis by converting glucose to glucose-6-phosphate. Our Co-IP and confocal microscopy results confirmed that there is a physical interaction between STING and HK_2_ in the cytoplasm, which was maintained during PRRSV infection (Figure 5C,D). This interaction is critical for STING-mediated glycolytic inhibition, as HK_2_ overexpression did not affect STING protein levels (Figure 4G,H), indicating that STING acts upstream of HK_2_. Although our current data does not provide direct mechanistic evidence for how STING impairs HK_2_ activity, our results, combined with published literature, support a model wherein STING directly binds to HK_2_ to suppress its enzymatic activity. A recent study showed that STING binds to the N-terminal mitochondrial-binding domain of HK_2_, which is adjacent to its catalytic domain. This binding is thought to sterically hinder glucose access to HK_2_’s catalytic site, thereby reducing its enzymatic activity [11]. Additionally, our confocal microscopy experiments show that STING and HK_2_ co-localize in the cytoplasm, rather than in mitochondria, and demonstrated that STING inhibits HK_2_ by disrupting its association with mitochondria [11].

The functional significance of the STING-HK_2_ axis in PRRSV replication was further validated using pharmacological and genetic approaches. Treatment with the HK_2_ inhibitor 2-DG reduced lactate levels and viral replication, while the glycolysis activator PS48 had the opposite effect (Figure 2A–G). Moreover, HK_2_ overexpression enhanced PRRSV N protein expression (Figure 2I,K), confirming that HK_2_ is a positive regulator of PRRSV replication. Collectively, these data demonstrate that PRRSV disrupts the STING-HK_2_ interaction by degrading STING, thereby unleashing HK_2_ activity to drive glycolysis and support viral replication. Notably, the STING-HK_2_-glycolysis axis may represent a common target for viruses that rely on aerobic glycolysis. For example, influenza A virus also upregulates HK_2_ and glycolysis to promote replication [23]. Thus, targeting this axis could have broad antiviral implications. Our results suggest that HK_2_ inhibitors (e.g., 2-DG) might serve as potential therapeutic agents, as 2-DG suppressed PRRSV replication by inhibiting glycolysis (Figure 2C).

While our findings highlight the STING-HK_2_ axis as a critical regulator of PRRSV replication, The molecular mechanism by which PRRSV degrades STING requires further investigation. PRRSV may utilize viral proteases (e.g., Nsp4 or Nsp7) to cleave STING [24], or it may upregulate host E3 ubiquitin ligases to promote STING degradation via the proteasome pathway. Additionally, in vivo studies using animal models are also needed to validate the therapeutic potential of targeting the STING-HK_2_ axis.

In conclusion, our study identifies a novel mechanism by which PRRSV degrades STING and then promotes HK_2_ expression, thereby activating glycolysis, promoting lactate accumulation. These findings reveal an intricate interplay among innate immunity (STING), cellular metabolism (HK_2_/glycolysis), and viral replication, and they suggest that targeting the STING-HK_2_-glycolysis axis may provide new strategies for PRRSV control. This work also expands our understanding of STING as a metabolic checkpoint, highlighting its dual role in antiviral defense and cellular metabolism regulation.

## Figures and Tables

**Table 1:** The siRNA sequences targeting the monkey STING gene.

siRNA Name	Primer Sequences (5′–3′)
STING-374-F	GCCUUUCGCAGGCACUGAATT
STING-374-R	UUCAGUGCCUGCGAAAGGCTT
STING-530-F	CCCGGAUUCAAACUUACAATT
STING-530-R	UUGUAAGUUUGAAUCCGGGTT
NC-F	UUCUCCGAACGUGUCACGUTT
NC-R	ACGUGACACGUUCGGAGAATT

## Data Availability

All data generated for this study are included in the article.

## Supplementary Materials

The following supporting information can be downloaded at: https://www.mdpi.com/article/10.3390/v18030284/s1.
