# Supplementary figures and images for: STING Degradation by PRRSV Activates HK2-Mediated Glycolysis to Facilitate Viral Replication
Source: Viruses. 2026 Feb 27;18(3):284. doi: 10.3390/v18030284 (PMC13030658; doi:10.3390/v18030284)

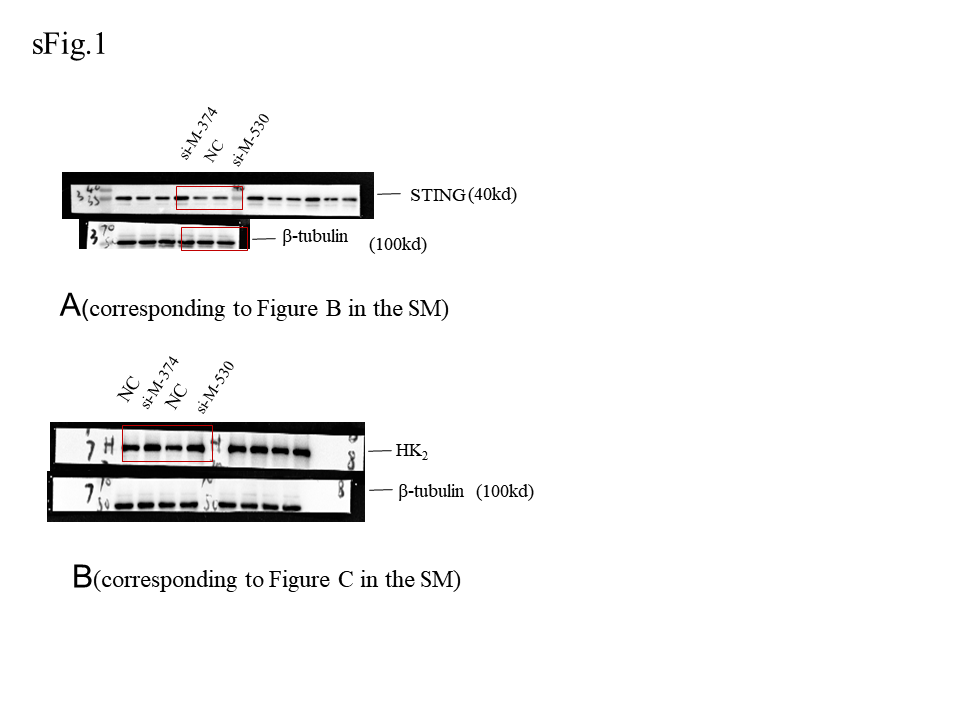

Supplement: Supplementary file 1 [file viruses-18-00284-s001.zip › viruses-4097895-supplementary-figureS1.tif]
